# The shifting burden of mortality among men with HIV in Japan between 2007 and 2024: a single-center retrospective cohort study

**DOI:** 10.1186/s12879-025-12114-8

**Published:** 2025-12-05

**Authors:** Keiji Konishi, Tomoko Uehira, Kazuyuki Hirota, Takashi Ueji, Yasuharu Nishida, Takuma Shirasaka, Dai Watanabe

**Affiliations:** 1 AIDS Medical Center, NHO Osaka National Hospital, 2-1-14 Hoenzaka, Chuo-ku, Osaka, 540-0006 Japan; 2 Department of Post-Infectious Disease Therapeutics, Graduate School of Medicine, The University of Osaka, Osaka, Japan; 3 Department of Advanced Medicine for HIV Infection, Graduate School of Medicine, The University of Osaka, Osaka, Japan

**Keywords:** HIV infections, Aged, Standardized mortality ratio, Neoplasms, Retrospective studies, Antiretroviral therapy

## Abstract

**Background:**

Antiretroviral therapy (ART) has transformed HIV infection from a fatal disease into a chronic condition and improved the life expectancy of people living with HIV (PLWH). Few studies have examined long-term changes in standardized mortality ratios (SMRs) by age and cause of death among PLWH in Japan. This study investigated the changes in SMR among men with HIV infection in Japan over an 18-year period, focusing on age at death and the contribution of malignancies.

**Methods:**

We conducted a retrospective cohort study of men with HIV infection who received care at Osaka National Hospital between 2007 and 2024. Data were extracted from medical records. Causes of death were classified according to the Coding Causes of Death in HIV protocol. SMRs were calculated based on the general male population of Japan.

**Results:**

A total of 3,793 patients were included with 35,007 person-years of follow-up. The median age on enrollment was 36.3 years, and the median follow-up period was 10.1 years. Of the patients, 230 died during the study period, with a median age at death of 53.4 years. The causes of death included 44 deaths from AIDS-defining illnesses, 20 deaths from AIDS-defining malignancies, 51 deaths from non-AIDS-defining malignancies; and 57 deaths of unknown cause. The SMR decreased from 4.11 in 2007–2011 to 1.27 in 2021–2024, with different patterns in different age groups. In recent years, the SMR in younger and older age groups approached that of the general population, but remained high in the 40–64-years age group, and in patients with CD4 counts < 200 cells/μL or viral loads ≥1000 copies/mL within the calendar year. The SMR for malignancies also remained high in the 40–64-years age group (2.28 in 2021–2024).

**Conclusions:**

Among men with HIV infection in Japan, the risk of death has declined markedly with the widespread use of ART, approaching that of the general population, particularly in younger and older age groups. However, elevated mortality due to non-AIDS-related causes, particularly malignancies, persists among middle-aged men. Future HIV care should prioritize viral suppression and enhancing age-appropriate cancer screening.

**Supplementary Information:**

The online version contains supplementary material available at 10.1186/s12879-025-12114-8.

## Background

Human immunodeficiency virus (HIV) infection was first recognized as a fatal disease in the 1980s. However, improvements in antiretroviral therapy (ART) have transformed HIV infection into a manageable chronic condition, leading to an improvement in life expectancy [1]. Globally, cohort studies from several countries have shown a decline in standardized mortality ratios (SMRs) with the widespread use of treatment, and survival now approaches that of the general population [2, 3]. Conversely, mortality rates from age-related chronic comorbidities have increased in people living with HIV (PLWH) [4–6], with more than 50% of PLWH dying from non-communicable chronic diseases [7–10]. However, markedly elevated SMRs have been observed among injection drug users and individuals who experienced delays in diagnosis and the initiation of treatment, revealing major disparities in life expectancy among different risk groups [11].

In Japan, where life expectancy ranks among the highest worldwide, the aging of the PLWH population presents unique challenges. The demographic shift is consistent with that reported in other countries [12–14], and in 2023, more than 10% of individuals with newly diagnosed HIV infection were aged 50 years or older [15]. This demographic shift has raised concerns about a possible increasing contribution of non-AIDS-defining malignancies (NADMs) to mortality among PLWH [16]. Previous Japanese studies have shown that elevated mortality persists among middle-aged and older PLWH, and those with a CD4 count < 200 cells/μL at time of diagnosis, with a low CD4 count being identified as an independent risk factor for all-cause mortality [17, 18]. However, few studies have comprehensively examined the long-term changes in SMR by age group and cause of death, including the impact of recent advancements, such as the introduction of integrase inhibitor-based ART, on the SMR. Although studies from other countries have highlighted the role of NADMs in contributing to elevated mortality among aging populations [11, 16], data on the extent of this phenomenon in men with HIV in Japan remain limited, highlighting the need to re-examine mortality patterns and disparities in prognosis among men with HIV in Japan in the context of improvements in ART.

Therefore, this study aimed to assess temporal changes in SMRs by age group and cause of death among men with HIV who received care between 2007 and 2024 by comparing the observed number of deaths with the expected number of deaths in the general male population in Japan to enable the provision of targeted HIV care.

## Methods

### Study design and setting

To evaluate temporal changes in the SMRs and epidemiological characteristics of men with HIV owing to advancements in ART, we conducted a single-center retrospective cohort study at Osaka National Hospital. By the end of 2024, the hospital had provided care for a cumulative total of 4,261 patients with HIV, accounting for approximately 12% of all cases in Japan, thereby serving as a major tertiary care center in the country [15].

### Patients and study period

The study period was from January 1, 2007, to December 31, 2024. Men with HIV who received care at Osaka National Hospital, including both outpatient and inpatient services, were included. Patients who first visited the hospital for the first time during the study period, as well as those who started attending regular follow-up visits prior to January 1, 2007, and had at least one visit during 2007 were included. Female patients and those aged younger than 20 years at their first visit on or after January 1, 2007, were excluded. Female patients were excluded owing to their small number (approximately 4% of the cohort) during the study period, which limited statistical power to perform detailed stratified analyses. Because more than 95.0% of newly reported PLWH in Japan are men [15], focusing this study on men with HIV aligns with national public health priorities and does not markedly compromise the generalizability of the results.

This study was approved by the Institutional Review Board of Osaka National Hospital (approval no.25034) and conducted in accordance with the principles of the Declaration of Helsinki. The requirement for individual informed consent was waived because the study was retrospective, and the data were anonymized prior to analysis. Information about the study was posted on the hospital’s website, and an opt-out approach was employed, allowing patients to decline participation in the use of their data. No patients opted out during the study period.

### Data collection and variables

Data on age and sex, date of the first visit to the hospital, history of AIDS-defining illnesses, details of prescribed ART, plasma HIV-1 RNA levels (viral load), CD4 counts, assumed mode of infection, and date of death, were extracted from medical records. In this study, “at enrollment” refers to the beginning of the follow-up period, defined as the date of the first visit on or after January 1, 2007. The lowest CD4 count and highest viral load values recorded in each calendar year were used as time-varying measures for that year’s analysis. Two HIV care specialists independently reviewed the medical records of patients who had died and classified the cause of death using the Coding Causes of Death in HIV (CoDe) protocol [19, 20]. In cases of disagreement, a third reviewer made the final classification. Deaths due to malignancies were confirmed by a histopathological diagnosis. Causes of death were classified as AIDS-related (codes 01.1, 01.2, 01) or non-AIDS-related (codes 02–92) according to the CoDe protocol. The follow-up began on the first hospital visit on or after January 1, 2007, and ended at the earliest of the following: the date of death, the date of the last visit (in patients lost-to-follow-up or referred to another institution), or December 31, 2024. Lost-to-follow-up was defined as no visits for more than 12 months after the last visit, with no confirmation of continued care elsewhere or of death. As part of the established patient follow-up system at our hospital, a coordinator nurse contacted patients who missed appointments to encourage them to return for scheduled visits.

### Statistical analysis

The SMR was used as the primary outcome measure. Person-years of observation were calculated for each patient, and each patient’s observation time was allocated to four predefined calendar periods (2007–2011, 2012–2016, 2017–2020, and 2021–2024). Deaths were analyzed according to the calendar year in which they occurred. SMRs were calculated by dividing the observed number of deaths in the study population by the expected number of deaths in the Japanese general male population, stratified by 5-year age intervals, based on official national vital statistics [21]. Endpoints included the annual SMRs (for all-cause and malignancy-related mortality), annual SMRs stratified by the CD4 count and viral load, and age group-specific SMRs (aggregated for 2007–2024). The calculation of 95% confidence intervals (CIs) for SMRs was performed under the assumption that deaths followed a Poisson distribution. To assess the impact of treatment, ART was treated as a time-dependent exposure in the analysis. Each patient’s person-time was longitudinally allocated to one of four mutually exclusive treatment statuses based on their treatment records: integrase strand transfer inhibitor (INSTI)-based, protease inhibitor (PI)-based, and non-nucleoside reverse transcriptase inhibitor (NNRTI)-based ART regimens, and “ART interruption/untreated.” The latter included periods with no anchor agent, NRTI-only regimens, or with missing treatment data. Observed deaths were attributed to the ART status on the date of death. SMRs were then calculated for each ART status. To formally test for temporal trends in SMRs, a p for trend was calculated using Poisson regression models. All statistical analyses were performed using R version 4.5.1 (The R Foundation for Statistical Computing, Vienna, Austria).

## Results

**Fig. 1:**
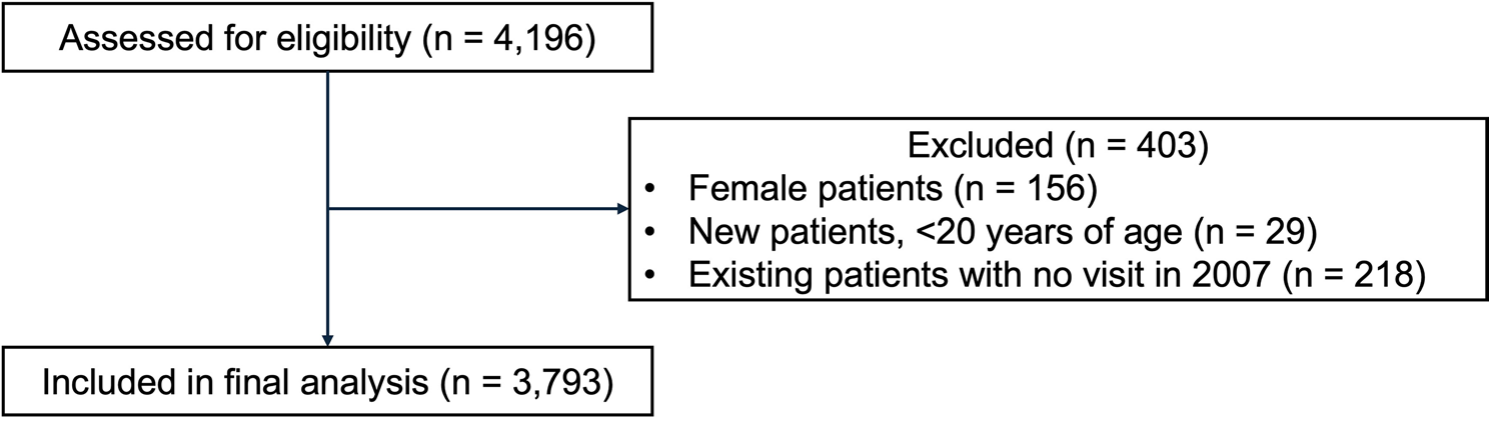
Flow chart showing participant selection

Of 4,196 patients assessed for eligibility, 3,793 who received care at Osaka National Hospital between 2007 and 2024 were included in the analysis (Fig. 1), with a median follow-up period of 10.1 years (interquartile range [IQR]: 4.1–15.5 years) and a cumulative follow-up of 35,007 person-years. The clinical characteristics of the participants are summarized in Table 1. At enrollment, the median age was 36.3 years (IQR: 29.7–44.9 years), the median CD4 count was 274 cells/μL (IQR: 113–420 cells/μL), and the median viral load was 4.7 log_10_ copies/mL (IQR: 3.8–5.3 log_10_ copies/mL). Of the 3,793 patients, 828 (21.8%) had a history of AIDS. The most common route of infection was male-to-male sexual contact (3,122 participants; 82.3%), and the majority of participants were of Japanese nationality (3,553 participants; 93.7%).

**Table 1:** Characteristics of study participants (*n* = 3793)

Variable	Value
Total person-years	35,007 person-years
Age at enrollment (years), median (IQR)	36.3 (29.7–44.9)
Japanese nationality, n (%)	3553 (93.7%)
CD4 count at enrollment (cells/μL), median (IQR)	274 (113–420)
HIV RNA level at enrollment (log_10_ copies/mL), median (IQR)	4.7 (3.8–5.3)
History of AIDS-defining illness, n (%)	828 (21.8%)
Route of transmission	
Male-to-male sexual contact	3122 (82.3%)
Heterosexual contact	439 (11.6%)
Contaminated blood products	69 (1.8%)
Injection drug use	5 (0.1%)
Other/Unknown	158 (4.2%)
Follow-up duration (years), median (IQR)	10.1 (4.1–15.5)

IQR, interquartile range

During the study period, 230 deaths were confirmed. The median age at death was 53.4 years (IQR: 45.6–64.7 years), and the median time from the first visit to death was 5.0 years (IQR: 0.9–10.7 years) (Table 2). The causes of death were diverse: 44 AIDS-related deaths, 20 due to AIDS-defining malignancies, 51 due to NADMs, and 57 from unknown causes. Forty-three deaths occurred outside healthcare institutions, of which 31 were from unknown causes. The detailed causes of death by age group are presented in the additional file (Table S1). In the 40–64-years age group, NADMs were the most common cause of death (33 cases), followed by AIDS-related deaths (29 cases) and non-malignant causes, such as cardiovascular disease (10 cases).

**Table 2:** Characteristics of deceased participants (*N* = 230)

Variable	Value
Age at death (years), median (IQR)	53.4 (45.6–64.7)
Time from the first visit to death (years), median (IQR)	5.0 (0.9–10.7)
Death during treatment interruption, n (%)	4 (1.7%)
Place of death, n (%)	
In-hospital	105 (45.7%)
Other hospital	49 (21.3%)
At home	33 (14.3%)
Outside hospital/Others	43 (18.7%)
Cause of death, n (%)	
AIDS-related (excluding malignancies)	44 (19.1%)
*Pneumocystis* pneumonia	11 (4.8%)
Progressive multifocal leukoencephalopathy	10 (4.3%)
HIV encephalopathy	6 (2.6%)
Cytomegalovirus infection	3 (1.3%)
Cryptococcal meningitis	2 (0.9%)
Others	12 (5.2%)
AIDS-defining malignancies	20 (8.7%)
Malignant lymphoma	19 (8.3%)
Kaposi’s sarcoma	1 (0.4%)
Non-AIDS-defining malignancies	51 (22.2%)
Lung cancer	14 (6.1%)
Gastric cancer	5 (2.2%)
Anal canal cancer	5 (2.2%)
Hepatocellular carcinoma	5 (2.2%)
Hematologic malignancy	5 (2.2%)
Colorectal cancer	3 (1.3%)
Oropharyngeal cancer	3 (1.3%)
Hilar cholangiocarcinoma	2 (0.9%)
Others	9 (3.9%)
Hepatitis C virus-related	4 (1.7%)
Cardiovascular disease	15 (6.5%)
Respiratory disease	9 (3.9%)
Suicide	9 (3.9%)
COVID-19	2 (0.9%)
Others	19 (8.3%)
Unknown	57 (24.8%)

IQR, interquartile range

The changes in the crude mortality rates for deaths from all causes and malignancy-related deaths are shown in the additional file (Table S2). The overall crude mortality rate for deaths from all causes over the entire observation period was 7.01 per 1,000 person-years. The highest rate (10.58 per 1,000 person-years) was observed in the 2007–2011 period, followed by a decrease to 5.27 per 1,000 person-years in 2017–2020. In the most recent period (2021–2024), the rate increased slightly to 5.80 per 1,000 person-years. The crude mortality rate for malignancy-related deaths during the whole observation period was 2.16 per 1,000 person-years. The crude mortality rates for malignancy-related deaths were 2.78, 2.39, 1.76, and 1.97 per 1,000 person-years in 2007–2011, 2012–2016, 2017–2020, and 2021–2024, respectively. Temporal changes in the crude mortality rates for deaths from causes other than malignancy are shown in the additional file (Table S3). The crude mortality rate for AIDS-related deaths decreased markedly from 3.53 per 1,000 person-years in 2007–2011 to 0.44 per 1,000 person-years in 2021–2024. Consequently, the relative importance of non-AIDS-related causes increased. During the most recent period (2021–2024), the mortality rates for respiratory diseases (0.55 per 1,000 person-years) and cardiovascular diseases (0.55 per 1,000 person-years) exceeded that for AIDS-related deaths. During this period, two deaths due to COVID-19 (0.22 per 1,000 person-years) were reported, and the mortality rate from suicide decreased.

**Fig. 2:**
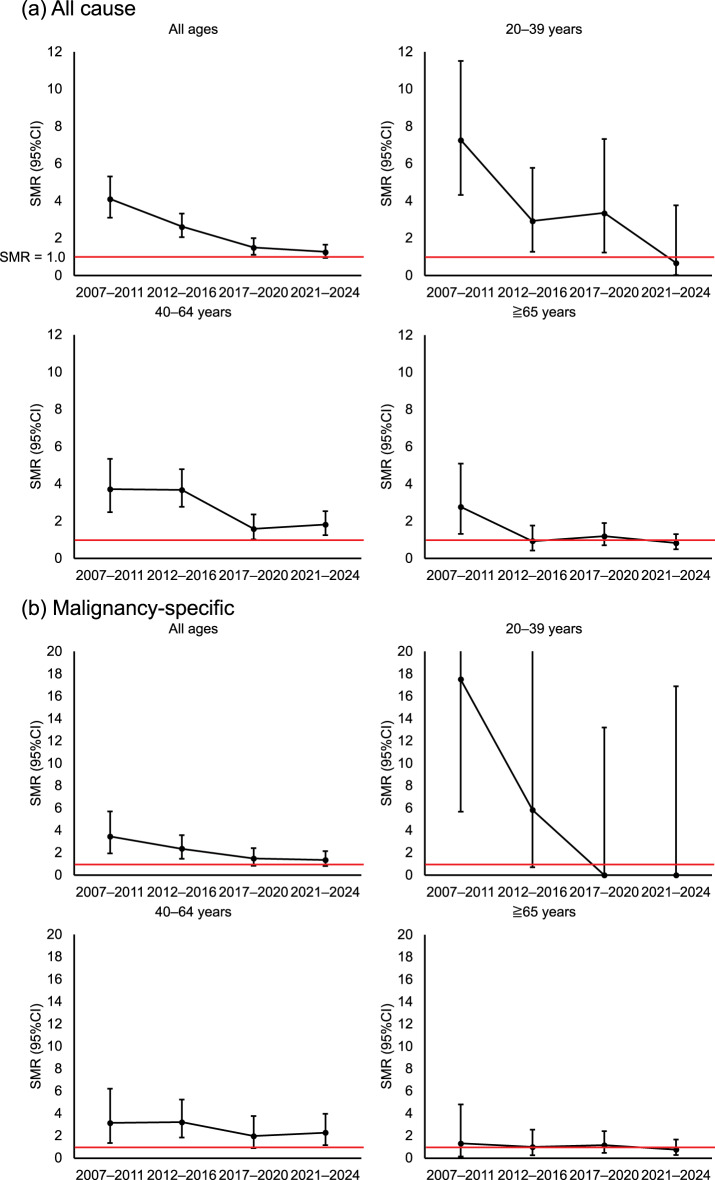
Standardized mortality ratios (SMRs) by period according to age group. (**a**) All-cause SMRs by period in the whole cohort and by age group. (**b**) Malignancy-specific SMRs in the whole cohort and by age group. The error bars show the 95% confidence intervals (CIs). The 95% CIs are wide in the 20–39-years age group owing to the limited number of deaths. The red horizontal line indicates an SMR of 1.0. An SMR of 0 indicates that there were no deaths in the specific subgroup during the specific period

The overall SMR declined markedly during the study period (Fig. 2) from 4.11 (95% CI: 3.11–5.32) in the 2007–2011 to 1.27 (95% CI: 0.95–1.66) in 2021–2024. In the 20–39-years age group, the SMR decreased from 7.28 (95% CI: 4.32–11.51) to a level that did not differ significantly from that of the general population (SMR 0.68, 95% CI: 0.02–3.77). In addition, the SMR decreased from 3.72 (95% CI: 2.49–5.35) to 1.82 (95% CI: 1.26–2.54) in the 40–64-years age group, and from 2.77 (95% CI: 1.33–5.10) to 0.83 (95% CI: 0.49–1.31) in the ≥65-years age group, and the SMR in the in the ≥65-years age group did not differ significantly from that of the general population. The SMR for malignancy-related deaths also decreased in all age groups, from 3.46 (95% CI: 1.94–5.71) to 1.36 (95% CI: 0.81–2.15). However, the malignancy-related SMR remained higher in the 40–64-years age group than in the younger and older age groups, decreasing from 3.15 (95% CI: 1.36–6.20) to 2.28 (95% CI: 1.18–3.98). Notably, in the 20–39-years age group, the malignancy-related SMR was 0 in the 2017–2020 and 2021–2024 periods. In the ≥65-years age group, the malignancy-related SMR was 0.77 (95% CI: 0.28–1.68) in the 2021–2024 period.

**Fig. 3:**
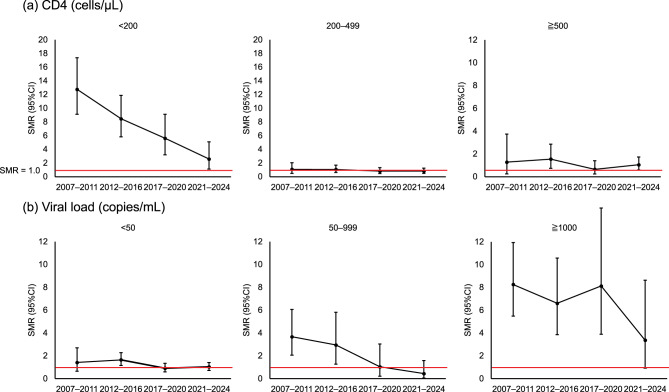
Standardized mortality ratios (SMRs) by period according to the time-varying lowest CD4 count and highest viral load level within each calendar year. (**a**) SMRs by period according to lowest CD4 count level within each calendar year. (**b**) SMRs by period according to highest viral load level within each calendar year. The error bars show the 95% confidence intervals (CIs). The red horizontal line indicates an SMR of 1.0

The changes in SMR stratified by the CD4 count and viral load, are shown in Fig. 3. For this analysis, each patient’s person-time contribution was assessed annually based on the lowest CD4 count or highest viral load level recorded within the calendar year. Among patients with CD4 counts < 200 cells/μL, the SMR was highest in the 2007–2011 period (12.76, 95% CI: 9.12–17.38) and decreased to 2.59 (95% CI: 1.12–5.11) in the 2021–2024 period. In contrast, patients with CD4 counts of 200–499 cells/μL and ≥500 cells/μL had lower SMRs of 0.82 (95% CI: 0.50–1.26) and 1.05 (95% CI: 0.59–1.74), respectively, in the 2021–2024 period. Similarly, among patients with viral loads ≥1,000 copies/mL, the SMR decreased from 8.26 (95% CI: 5.49–11.93) in the 2007–2011 period to 3.37 (95% CI: 0.92–8.64) in the 2021–2024 period. In contrast, patients with viral loads < 50 copies/mL maintained low SMRs, which from 1.42 (95% CI: 0.65–2.70) in the 2007–2011 period to 1.03 (95% CI: 0.72–1.41) in the 2021–2024 period.

In the time-dependent analysis, the crude mortality rates (per 1,000 person-years) over the entire observation period (2007–2024) were 6.01 for INSTI-based ART, 5.34 for NNRTI-based ART, 4.91 for PI-based ART, and 20.42 for ART interruption/untreated. The corresponding all-cause SMRs were 1.72 (95% CI, 1.42–2.06) for INSTI-based ART, 1.55 (0.97–2.34) for NNRTI-based ART, 1.57 (1.13–2.14) for PI-based ART, and 10.43 (8.41–12.79) for ART interruption/untreated. Across the four calendar periods, SMRs generally declined within each ART class (Table S4).

In analyses stratified by AIDS status at enrollment, the crude mortality rates (per 1,000 person-years) and SMRs declined over calendar time both in patients with and without an AIDS-defining illness at enrollment but remained consistently higher in those with an AIDS-defining illness at enrollment (Table S5).

## Discussion

This study provides a detailed analysis of the long-term changes in SMRs and mortality patterns among a cohort of men with HIV who received care at a tertiary medical center in Osaka between 2007 and 2024. The marked decrease in overall SMR, including a decrease in the mortality rate in the 20–39-years age group to a level approaching that of the general population, suggests that improvements in ART during the study period markedly improved the life expectancy of PLWH in Japan. However, the results of this study revealed that elevated mortality, particularly due to malignancies, persists in men in the 40–64-years age group.

The marked reduction in all-cause SMR can be attributed to earlier ART initiation and high rates of viral suppression. These results align with findings of a UK cohort study that demonstrated that a delayed diagnosis markedly shortened life expectancy [22], and an analysis of 23 European cohorts that showed that mortality rates in PLWH decreased by 50% during this period [3]. Collectively, these findings suggest that timely diagnosis of HIV infection and early treatment initiation are associated with improved survival, including in Japan. This study revealed that the SMR in men in the 20–39-years age group decreased to a level that did not differ significantly from that of the general population in the most recent period. This may reflect the benefits of frequent healthcare access, increased health literacy, and the rigorous management of lifestyle-related conditions. Improved life expectancy has also been reported among younger PLWH in the UK, based on an analysis of national surveillance data [23], suggesting that the current model of HIV care is effective in younger populations.

The median age at death in this cohort was 53.4 years with an IQR of 45.6–64.7 years, indicating that mortality was concentrated in individuals in the 40–64-years age group. This aligns with the finding that whereas the SMR in the 20–39-years age group fell below that of the general population, the mortality rate remained elevated in the 40–64-years age group. The clustering of deaths in this age group suggests that individuals who acquired HIV prior to the widespread availability of ART, and those who were diagnosed or initiated treatment late, may experience a greater burden of comorbidities and NADMs as they age, leading to increased mortality. A large proportion of deaths, including the majority of deaths from unknown causes, occurred outside a medical institution, which highlights a critical challenge in HIV care in Japan. Furthermore, the median time from the first hospital visit to death was 5.0 years, a relatively short period that likely reflects the adverse impact of late diagnosis on prognosis. In a large-scale cohort study conducted by Trickey et al. [3] in Europe and North America, suicide and accidental deaths were two of the leading causes of death. Although the mortality rate from suicide decreased in our cohort during the study period, the high number of out-of-hospital deaths due to unknown causes warrants further investigation. To gain a comprehensive understanding of mortality, collection of more detailed data on out-of-hospital deaths is essential. Among patients aged 65 years and older, the SMR decreased to 0.83, a level that did not differ significantly from that of the general population. This may be partly explained by the “healthy survivor effect,” whereby individuals who have survived through the pre-ART era and maintained long-term ART adherence may represent a healthier subpopulation in terms of self-management, healthcare access, and lifestyle behaviors. These individuals may be regarded as “selective survivors” who have passed through a natural selection process shaped by disease progression, potentially introducing a bias that leads to a lowering of the SMR in this age group [24]. Therefore, although the reduced SMR in older adults may reflect the long-term benefits of ART, it requires interpretation in light of possible survivorship bias. Future studies need to consider incorporating statistical models that account for survivorship bias and confirm the data obtained through multicenter collaborative research.

Among patients with CD4 counts < 200 cells/μL, the most recent SMR remained high (2.59). This at-risk population likely consists of patients with a late diagnosis and those with insufficient immune recovery (i.e., immunological non-responders) despite long-term ART. Our findings, which demonstrate a strong and persistent association between late presentation (an AIDS-defining illness at enrollment) and excess mortality, reinforce this concern. This is consistent with a prospective cohort study from Japan which reported that a delayed diagnosis doubled the risk of death [18], supporting the hypothesis that risk is not fully eliminated even after immune reconstitution. Although the SMR gap between those with and without AIDS at enrollment narrowed over time, the residual excess mortality among late presenters highlights the need for targeted interventions to reduce diagnostic delays. Elevated mortality in this group may be attributable not only to opportunistic infections and specific immunodeficiency-related malignancies, but also to the increased severity of NADMs. In Japan, obtaining the public subsidy certificate required to receive financial assistance for HIV treatment costs may take several months [25]. Many patients delay ART initiation until the certificate is issued, which may contribute to delays in initiating treatment. Although the SMR among those with viral loads ≥1,000 copies/mL was 3.37, this was not statistically significant owing to the small absolute number of patients in this group. This group is likely to have consisted primarily of patients with poor adherence to ART and those with treatment interruptions. This result suggests the negative impact of structural and behavioral factors, such as poor medication adherence, psychiatric disorders, and substance use, which are factors that have been identified in European multicenter reports as major contributors to disparities in mortality [11]. Furthermore, a large number of PLWH in Japan remain undiagnosed. A recent mathematical modeling study estimated that at the end of 2022, the proportion of PLWH diagnosed with HIV infection was lowest among those in their 30s (89.4%) [26]. The same study also identified a growing difficulty in diagnosing HIV infection in those aged 40 years and older, noting a decline in the annual diagnosis rate in this age group. This diagnostic gap, particularly in the middle-aged population, aligns with the finding of persistently elevated mortality in the 40–64-years age group in this study and highlights the urgent need for targeted interventions.

The persistently elevated malignancy-related mortality is likely due to a multifactorial interplay between chronic inflammation, immunosenescence, co-infection with oncogenic viruses, and lifestyle factors, such as smoking and alcohol consumption. A recent meta-analysis of NADM risk worldwide found the both NADM incidence and mortality are increasing [27]. Similarly, a prospective cancer-screening study among PLWH with hemophilia in Japan revealed a high prevalence and incidence of NADM, highlighting the hidden cancer burden and utility of regular screening [28]. The sustained elevation of the malignancy-related SMR in the 40–64-year age group in this study suggests a need for intensified surveillance in this population. A secondary analysis of the Strategic Timing of AntiRetroviral Treatment (START) trial, conducted in Europe and the United States, revealed that early ART initiation reduced the risk of infection-related cancers [29]. However, in Japan, approximately 30% of cases of HIV infection are diagnosed after the onset of AIDS [15], highlighting the need to address diagnostic delays and establish individualized cancer-screening programs. The meta-analysis of NADM by Yuan et al. [27], highlighted the importance of integrating primary and secondary prevention of major NADMs, such as anal, liver, and lung cancers, into routine HIV care. This includes age-stratified screening strategies that combine human papillomavirus and hepatitis B virus vaccination, antiviral therapy, smoking cessation support, and the use of screening methods, such as low-dose computed tomography and endoscopy. This study suggests that in an aging PLWH population, it is necessary to go beyond cancer screening and incorporate comprehensive geriatric assessment and mental health support into multidisciplinary team-based care. According to an analysis of Japanese health insurance claims data, 13.4% of 28,089 PLWH on ART were using five or more concurrent medications, with rates increasing to 29.9% in those aged 50–59 years and 40.3% in those aged 70 years and older [30]. Therefore, the systematic management of polypharmacy and drug-drug interactions is a growing challenge in the care of older PLWH.

This study has several strengths. The long-term follow-up of a cohort of almost 4,000 individuals at Osaka National Hospital, a central institution for PLWH care in Japan, over an 18-year-period, provides new insights into the epidemiology of HIV in Japan, and a comprehensive evaluation of the long-term changes in mortality risk. Additionally, the use of the CoDe protocol for cause-of-death classification ensured internationally comparable and standardized cause-of-death data, enhancing the reliability of the results obtained. Furthermore, by conducting stratified analyses by age group, CD4 count, and viral load, the study identified age group-specific challenges, such as an improved prognosis in the younger age group, and persistently elevated mortality due to malignancies in the middle and older age groups.

Our time-dependent analyses reinforce two messages. First, mortality during ART interruption or untreated time remained substantially higher than the expected mortality of men in the general population, even though it decreased over calendar time, highlighting the importance of sustained retention in care and viral suppression. Second, SMRs within ART classes declined over time, reaching ~1.4 for INSTI in 2021–2024. Because ART classes changed over time and according to the stage of infection, these comparisons are descriptive and are subject to confounding by indication and time period; therefore, causal comparisons between ART classes should be avoided. Nevertheless, the results are consistent with the overall improvement in prognosis associated with modern ART and retention in care.

The SMR gap for patients with and without an AIDS-defining illness at enrollment narrowed over time from ~2.27 in 2007–2011 to 1.34 in 2021–2024, indicating improvements in prognosis but with persistent excess mortality among those with an AIDS-defining illness at enrollment. These findings demonstrate a strong and persistent association between late presentation (an AIDS-defining illness at enrollment) and excess mortality, with absolute SMRs decreasing over time in both patients with and without an AIDS-defining illness at enrollment. The narrowing SMR gap suggests improvements owing to earlier diagnosis, increased ART availability, and higher retention in care; however, the residual excess mortality among late presenters highlights the need for targeted interventions to reduce diagnostic delays, ensure early ART initiation, and retain patients in care.

This study has several limitations. Because it is a retrospective single-center cohort study, the possibility of a selection bias, such as overrepresentation of patients with more severe disease owing to referrals, cannot be ruled out. Therefore, the mortality rates and cause-of-death patterns may not be generalizable to all men with HIV in Japan. Furthermore, the cause of death was unknown in approximately 25% of all deaths and was higher among patients with out-of-hospital deaths. The high proportion of deaths due to unknown causes may have affected the accuracy of cause-specific SMRs, especially for non-AIDS-related deaths, and may have led to an underestimation of malignancy-related mortality. Although the precise causes of death remain undetermined as many of these deaths occurred out-of-hospital without autopsy, a review of medical records indicated that most of these individuals did not have documented severe comorbidities, such as ischemic heart disease or arrhythmias, that would predispose them to sudden cardiovascular death. Moreover, the minimum CD4 count and the maximum viral load within each year were used as immunological and virological markers, respectively but do not necessarily reflect long-term disease control at the individual level. To evaluate the effects of temporal fluctuations in CD4 count and viral load during the course of HIV infection, longitudinal analyses or analyses using time-weighted averages are required. Another limitation is that this study excluded female patients, and the cohort was predominantly composed of men who acquired HIV through male-to-male sexual contact (approximately 80%); therefore, differences in mortality by sex and route of transmission could not be assessed. Addressing this limitation is important for future research, particularly from a public health perspective of promoting diversity and inclusion. In addition, owing to the retrospective nature of the study and reliance on medical records, it was not possible to systematically capture social factors that affect health, such as smoking, alcohol use, lifestyle behaviors, other coinfections, comorbidities, immunological markers such as the CD4/CD8 ratio, mental health conditions, and use of medical cost subsidies. Although the rising incidence of syphilis among PLWH is a global concern, a recent nationwide Japanese study suggested the prevalence of syphilis was not increasing among PLWH in Japan [31]. However, our study did not systematically capture data on coinfections such as syphilis so this is an area for future research. The impact of these factors on mortality and prognosis need to be examined in future prospective studies or nationwide database analyses.

## Conclusions

This study revealed that SMRs among men with HIV in Japan improved markedly between 2007 and 2024, which is primarily due to improvements in ART. Although the mortality rates in the younger and older age groups converged toward those observed in the general population, mortality related to malignancies remained elevated in men in the 40–64-years age group. These results highlight the need for a comprehensive approach to NADMs in addition to virological control in providing care for PLWH in Japan and could inform the development of targeted public health and clinical management strategies.

## Electronic supplementary material

Below is the link to the electronic supplementary material.

Supplementary Material 1

## Data Availability

The data used in this study cannot be posted in supplementary files or public repositories because of legal and ethical restrictions.

## Abbreviations

AIDS: Acquired immunodeficiency syndrome
ART: Antiretroviral therapy
HIV: Human immunodeficiency virus
INSTI: Integrase strand transfer inhibitor
NADM: Non-AIDS-defining malignancy
NNRTI: Non-nucleoside reverse transcriptase inhibitor
PI: Protease inhibitor
PLWH: Persons living with HIV
SMR: Standardized mortality ratio
