# Supplementary material for: The shifting burden of mortality among men with HIV in Japan between 2007 and 2024: a single-center retrospective cohort study
Source: BMC Infect Dis. 2025 Dec 5;25:1694. doi: 10.1186/s12879-025-12114-8 (PMC12681175; doi:10.1186/s12879-025-12114-8)
Supplement: Supplementary file 1 — Supplementary Material 1 [file 12879_2025_12114_MOESM1_ESM.docx]

**The shifting burden of mortality among men with HIV in Japan between 2007 and 2024: A single-center retrospective cohort study**

**Additional file (supplementary tables)**

**Table S1.** Causes of death among men with HIV by age group, 2007–2024

**Table S2.** Crude mortality rates and standardized mortality ratios (SMR) of men with HIV compared with men in the general population in Japan

**Table S3.** Number of deaths and crude mortality rates (per 1,000 person-years) for causes of death other than malignancies, stratified by study period

**Table S4.** Time-dependent ART class-specific crude mortality rates and SMRs, overall and by period

**Table S5.** Crude mortality rates and SMRs by AIDS status at enrollment, overall and by period

Table S1. Causes of death among men with HIV by age group, 2007–2024

| Cause of death | Age group (years) | | |
| --- | --- | --- | --- |
|  | 20–39 | 40–64 | ≥65 |
| AIDS-related deaths | 8 | 29 | 7 |
| AIDS-defining malignancies | 4 | 12 | 4 |
| Non-AIDS-defining malignancies | 2 | 33 | 16 |
| Hepatitis C virus | 0 | 4 | 0 |
| Cardiovascular disease | 1 | 10 | 4 |
| Respiratory disease | 0 | 3 | 8 |
| Suicide | 4 | 4 | 1 |
| Others/unknown | 8 | 50 | 18 |

Table S2. Crude mortality rates and standardized mortality ratios (SMRs) in men with HIV compared with men in the general population of Japan

|  | Entire period | 2007–2011 | 2012–2016 | 2017–2020 | 2021–2024 | P for trend |
| --- | --- | --- | --- | --- | --- | --- |
| All causes |  |  |  |  |  |  |
| Crude mortality rate (/1,000 PY) | 7.01 | 10.58 | 7.83 | 5.27 | 5.8 |  |
| SMR (95% CI) | 2.00 (1.75–2.28) | 4.11 (3.11–5.32) | 2.63 (2.06–3.32) | 1.51 (1.11–2.00) | 1.27 (0.95–1.66) | <0.001 |
| **By age group** |  |  |  |  |  |  |
| Age group: 20–39 years |  |  |  |  |  |  |
| Crude mortality rate (/1,000 PY) | 2.86 | 6.16 | 2.14 | 2.19 | 0.46 |  |
| SMR (95% CI) | 3.9 (2.68–5.48) | 7.28 (4.32–11.51) | 2.93 (1.27–5.78) | 3.36 (1.23–7.32) | 0.68 (0.02–3.77) | 0.007 |
| Age group: 40–64 years |  |  |  |  |  |  |
| Crude mortality rate (/1,000 PY) | 7.45 | 12.63 | 10.94 | 4.25 | 5.58 |  |
| SMR (95% CI) | 2.51 (2.12–2.96) | 3.72 (2.49–5.35) | 3.68 (2.77–4.79) | 1.59 (1.02–2.37) | 1.82 (1.26–2.54) | <0.001 |
| Age group: ≥65 years |  |  |  |  |  |  |
| Crude mortality rate (/1,000 PY) | 25.15 | 63.50 | 20.89 | 25.19 | 20.36 |  |
| SMR (95% CI) | 1.10 (0.83–1.43) | 2.77 (1.33–5.10) | 0.93 (0.43–1.77) | 1.20 (0.71–1.90) | 0.83 (0.49–1.31) | 0.022 |
| **Malignancies** |  |  |  |  |  |  |
| Crude mortality rate (/1,000 PY) | 2.16 | 2.78 | 2.39 | 1.76 | 1.97 |  |
| SMR (95% CI) | 1.89 (1.47–2.38) | 3.46 (1.94–5.71) | 2.36 (1.48–3.58) | 1.49 (0.85–2.42) | 1.36 (0.81–2.15) | 0.004 |
| **Malignancies by age group** |  |  |  |  |  |  |
| Age group: 20–39 years |  |  |  |  |  |  |
| Crude mortality rate (/1,000 PY) | 0.61 | 1.71 | 0.54 | 0 | 0 |  |
| SMR (95% CI) | 6.78 (2.72–13.96) | 17.51 (5.68–40.85) | 5.83 (0.71–21.04) | 0.00 (0.00–13.20) | 0.00 (0.00–16.90) | 0.032 |
| Age group: 40–64 years |  |  |  |  |  |  |
| Crude mortality rate (/1,000 PY) | 2.36 | 3.48 | 3.18 | 1.59 | 1.97 |  |
| SMR (95% CI) | 2.6 (1.9–3.48) | 3.15 (1.36–6.20) | 3.22 (1.84–5.23) | 1.98 (0.91–3.76) | 2.28 (1.18–3.98) | 0.286 |
| Age group: ≥65 years |  |  |  |  |  |  |
| Crude mortality rate (/1,000 PY) | 8.69 | 12.7 | 9.28 | 9.8 | 6.79 |  |
| SMR (95% CI) | 0.99 (0.59–1.54) | 1.33 (0.16–4.81) | 1.00 (0.27–2.56) | 1.17 (0.47–2.41) | 0.77 (0.28–1.68) | 0.509 |
| **CD4 count (cells/µL)** |  |  |  |  |  |  |
| <200 |  |  |  |  |  |  |
| Crude mortality rate (/1,000 PY) | 32.52 | 46.01 | 35.25 | 24.65 | 15.15 |  |
| SMR (95% CI) | 7.48 (6.07–9.13) | 12.76 (9.12–17.38) | 8.47 (5.83–11.89) | 5.62 (3.21–9.13) | 2.59 (1.12–5.11) | <0.001 |
| 200–499 |  |  |  |  |  |  |
| Crude mortality rate (/1,000 PY) | 3.47 | 2.65 | 3.33 | 3.31 | 4.42 |  |
| SMR (95% CI) | 0.91 (0.70–1.16) | 1.08 (0.50–2.05) | 1.06 (0.63–1.68) | 0.82 (0.47–1.32) | 0.82 (0.50–1.26) | 0.342 |
| ≥500 |  |  |  |  |  |  |
| Crude mortality rate (/1,000 PY) | 2.94 | 2.77 | 3.55 | 1.67 | 3.68 |  |
| SMR (95% CI) | 1.05 (0.73–1.47) | 1.28 (0.26–3.74) | 1.55 (0.75–2.86) | 0.65 (0.24–1.41) | 1.05 (0.59–1.74) | 0.444 |
| **HIV viral load (copies/mL)** |  |  |  |  |  |  |
| <50 |  |  |  |  |  |  |
| Crude mortality rate (/1,000 PY) | 4.45 | 4.4 | 5.28 | 3.3 | 4.86 |  |
| SMR (95% CI) | 1.16 (0.95–1.41) | 1.42 (0.65–2.7) | 1.64 (1.15–2.27) | 0.91 (0.59–1.34) | 1.03 (0.72–1.41) | 0.058 |
| 50–999 |  |  |  |  |  |  |
| Crude mortality rate (/1,000 PY) | 6.2 | 9.80 | 8.69 | 3.29 | 1.74 |  |
| SMR (95% CI) | 1.97 (1.31–2.85) | 3.67 (2.06–6.06) | 2.95 (1.27–5.81) | 1.04 (0.22–3.04) | 0.44 (0.05–1.59) | <0.001 |
| >1000 |  |  |  |  |  |  |
| Crude mortality rate (/1,000 PY) | 14.24 | 15.76 | 12.05 | 16.93 | 10.95 |  |
| SMR (95% CI) | 7.04 (5.36–9.08) | 8.26 (5.49–11.93) | 6.61 (3.85–10.58) | 8.13 (3.90–14.96) | 3.37 (0.92–8.64) | 0.160 |

CI, confidence interval; PY, person-years; SMR, standardized mortality ratio

Table S3. Number of deaths and crude mortality rates (per 1,000 person-years) by causes of death other than malignancies, stratified by study period

| Study period | Causes of death | No. of deaths | Crude mortality rate (per 1,000 PY) |
| --- | --- | --- | --- |
| 2007–2011 | AIDS-related death | 19 | 3.53 |
|  | Suicide | 4 | 0.74 |
|  | Cardiovascular disease | 3 | 0.56 |
|  | Liver disease | 3 | 0.56 |
|  | Respiratory disease | 1 | 0.19 |
|  | Unknown/others | 12 | 2.23 |
| 2012–2016 | AIDS-related death | 16 | 1.74 |
|  | Cardiovascular disease | 3 | 0.33 |
|  | Suicide | 3 | 0.33 |
|  | Respiratory disease | 1 | 0.11 |
|  | Unknown/others | 27 | 2.94 |
| 2017–2020 | AIDS-related death | 5 | 0.55 |
|  | Cardiovascular disease | 4 | 0.44 |
|  | Suicide | 2 | 0.22 |
|  | Respiratory disease | 2 | 0.22 |
|  | Unknown/others | 19 | 2.09 |
| 2021–2024 | Respiratory disease | 5 | 0.55 |
|  | Cardiovascular disease | 5 | 0.55 |
|  | AIDS-related death | 4 | 0.44 |
|  | COVID‑19 | 2 | 0.22 |
|  | Liver disease | 1 | 0.11 |
|  | Unknown/others | 18 | 1.97 |

PY, person-years

Table S4. Time-dependent ART class-specific crude mortality rates and standardized mortality ratios (SMRs), overall and by period

|  | Entire period | 2007–2011 | 2012–2016 | 2017–2020 | 2021–2024 | P for trend |
| --- | --- | --- | --- | --- | --- | --- |
| INSTI-based |  |  |  |  |  |  |
| Crude mortality rate (/1,000 PY) | 6.01 | 8.99 | 7.39 | 5.21 | 5.81 |  |
| SMR (95% CI) | 1.72 (1.42–2.06) | 2.81 (0.77–7.19) | 2.54 (1.74–3.58) | 1.67 (1.18–2.30) | 1.39 (1.01–1.86) | <0.001 |
| NNRTI-based |  |  |  |  |  |  |
| Crude mortality rate (/1,000 PY) | 5.34 | 9.79 | 3.95 | 1.23 | 3.27 |  |
| SMR (95% CI) | 1.55 (0.97–2.34) | 3.87 (1.93–6.92) | 1.42 (0.46–3.31) | 0.31 (0.01–1.72) | 0.65 (0.13–1.89) | 0.068 |
| PI-based |  |  |  |  |  |  |
| Crude mortality rate (/1,000 PY) | 4.91 | 4.97 | 5.21 | 3.17 | 5.59 |  |
| SMR (95% CI) | 1.57 (1.13–2.14) | 1.97 (1.05–3.36) | 1.79 (1.04–2.87) | 0.87 (0.28–2.03) | 1.20 (0.39–2.79) | 0.228 |
| ART interruption/untreated |  |  |  |  |  |  |
| Crude mortality rate (/1,000 PY) | 20.42 | 14.12 | 11.94 | 8.56 | 11.15 |  |
| SMR (95% CI) | 10.43 (8.41–12.79) | 8.48 (5.68–12.18) | 7.46 (4.42–11.79) | 3.50 (1.14–8.16) | 2.56 (0.70–6.56) | <0.001 |

ART, antiretroviral therapy; CI, confidence interval; INSTI, integrase strand transfer inhibitor; NNRTI, non-nucleoside reverse transcriptase inhibitor; PI, protease inhibitor; PY, person years; SMR, standardized mortality ratio

Table S5. Crude mortality rates and standardized mortality ratios (SMRs) according to whether the patient had AIDS status at enrollment, overall and by period

|  | Entire period | 2007–2011 | 2012–2016 | 2017–2020 | 2021–2024 | P for trend |
| --- | --- | --- | --- | --- | --- | --- |
| History of AIDS-defining illness |  |  |  |  |  |  |
| Crude mortality rate (/1,000 PY) | 12.64 | 22.44 | 14.89 | 8.81 | 10.78 |  |
| SMR (95% CI) | 2.66 (2.18–3.21) | 6.41 (4.36–9.10) | 3.39 (2.35–4.74) | 1.70 (1.02–2.66) | 1.61 (1.02–2.42) | <0.001 |
| No history of AIDS-defining illness |  |  |  |  |  |  |
| Crude mortality rate (/1,000 PY) | 4.66 | 6.32 | 5.34 | 4.07 | 4.75 |  |
| SMR (95% CI) | 1.66 (1.39–1.98) | 2.82 (1.84–4.13) | 2.14 (1.52–2.94) | 1.37 (0.92–1.97) | 1.20 (0.83–1.68) | <0.001 |

CI, confidence interval; PY, person years; SMR, standardized mortality ratio
